# Layer-Dependent Oxidation Spreading in Multilayer Graphene during AFM Local Anodic Oxidation

**DOI:** 10.1021/acsomega.6c03252

**Published:** 2026-05-22

**Authors:** Jan Vymazal, Martin Konecny, Jakub Piastek, Jindrich Mach, Linda Supalová, Tomas Sikola, Miroslav Bartosik

**Affiliations:** † 232848 Central European Institute of Technology − Brno University of Technology (CEITEC BUT), Purkyňova 123, 612 00 Brno, Czech Republic; ‡ Institute of Physical Engineering, 613011 Brno University of Technology, Technická 2, 616 69 Brno, Czech Republic; § Department of Physics and Materials Engineering, Faculty of Technology, Tomas Bata University in Zlín, Zlín 760 01, Czech Republic

## Abstract

Local anodic oxidation is a practical method for creating graphene oxide nanostructure prototypes for fundamental research, such as in nanoelectronics. It is usually performed by scanning the atomic force microscopy (AFM) tip over an area designated for oxidation. The spreading of the oxidation reaction from a stationary tip has been rarely studied, despite its potential usefulness for fabricating graphene structures. While previous work investigated oxidation spreading on a graphene monolayer, this study extends the research to multilayer graphene to examine how the number of graphene layers affects the process. Depending on the number of layers, three pattern categories were identified. For 2–8 layers, “graphene craters” (a central hole surrounded by a raised circular rim) were observed. Beginning from 8 layers to 14 layers, “isotropic etching”, which removes a portion of the graphene layer in a relatively uniform manner, emerged. Finally, on 28-layer-thick graphene, irregular “anisotropic etching” was observed. Additionally, the increase of pattern radius with relative humidity was observed, whereas no clear dependence on exposure time was observed. The properties of the three categories of fabricated graphene patterns were characterized using AFM topography, Kelvin probe force microscopy, and Raman spectroscopy. The experiments were complemented by electric field calculations performed in COMSOL Multiphysics. By combining experimental results and simulations, a theoretical model was proposed to explain the formation of the observed patterns, based on oxidation initiated at defects and cleavages in the graphene.

## Graphical abstract

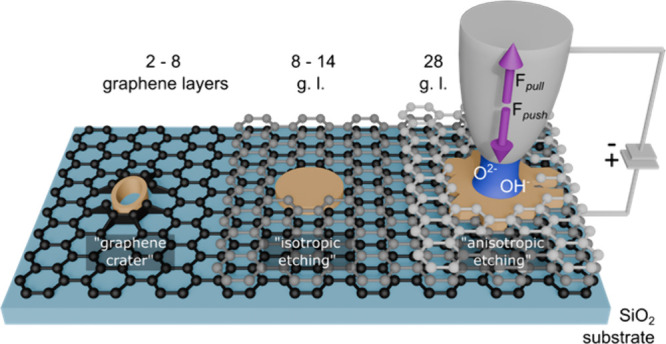

## 1 Introduction

Graphene is a well-known two-dimensional material that was first isolated in 2004. It is a crystal of carbon atoms arranged in a honeycomb lattice, possessing extraordinary electronic properties, such as high charge carrier mobility, low electronic noise, sensitivity to adsorbents, biocompatibility, and the ability to be patterned by lithographic methods. These properties make graphene a suitable material for manufacturing a wide range of electronic nanodevices.^1–7^ Local anodic oxidation (LAO), performed using atomic force microscopy (AFM), has become a convenient technique for the fabrication of prototype graphene nanoelectronic devices, enabling the creation of insulating barriers with nanometer dimensions or biosensors through the attachment of functional groups via graphene oxide.^4^

The traditional way of patterning graphene by LAO is based on oxidation along a line to create nanoribbons or trenches, interrupt graphene channels, or prepare graphene/graphene oxide/graphene junctions, among others.^3,8–17^ Alternatively, rectangular areas have been oxidized by scanning over predefined regions.^3,18–21^ However, in 2019, a third method was introduced,^22^ offering high reproducibility and circular shape of the oxidation spots, together with the ability to process square millimeters of graphene in a range of minutes. The method, schematically shown in Figure 1, is based on approaching the AFM tip to graphene until a single contact is established, followed by tip retraction while a constant voltage is applied throughout the process.^22^ High operating voltages (15–60 V) and relative humidity levels (69–99%) were employed, with exposure times ranging from 0.1 s (single touch) to 4 s (prolonged exposure). The radius of the oxidized area increased significantly with all these parameters.^22^

**Figure 1:**
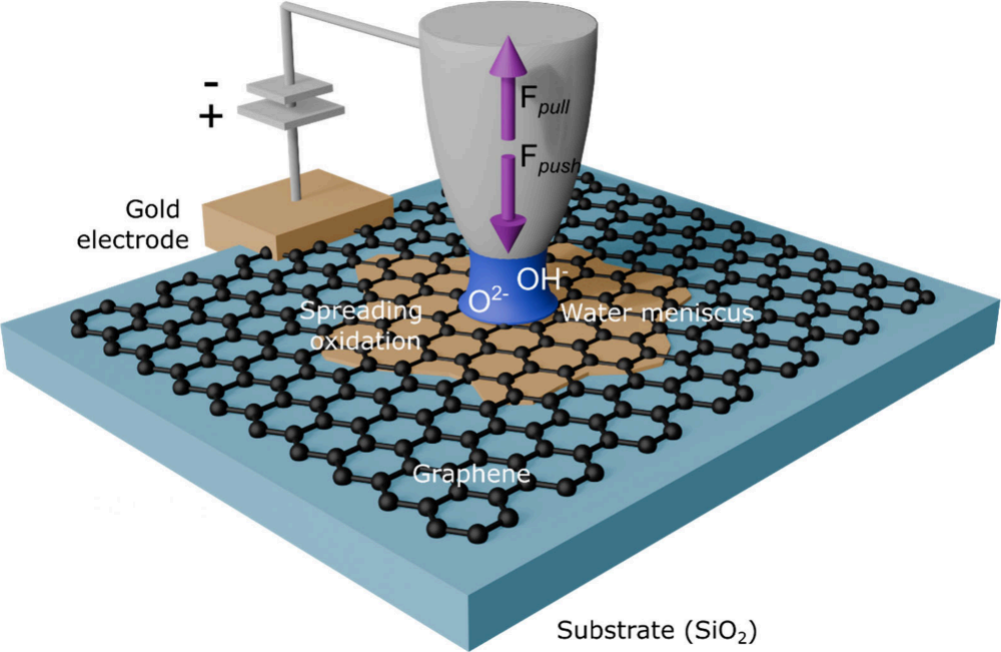
Schematic illustration of oxidation spreading induced by an AFM tip applied on a graphene layer on a SiO_2_ substrate. The negative bias voltage was applied between the tip and the graphene. The water meniscus, acting as a source of oxygen groups, forms under the tip at sufficient humidity. From the AFM tip, the oxidation spreads radially across the graphene layers, with the spreading depending on the number of graphene layers and the ambient humidity.

This paper extends previous research to graphene multilayers. The conventional parameters of humidity and exposure time are considered; however, particular emphasis is placed on the influence of the number of graphene layers. It is demonstrated that the number of graphene layers determines the basic characteristics of the prepared pattern (Section 3.4), while relative humidity governs the pattern size (Section 3.5). No clear dependence on the exposure time was found (Section 3.6). The oxidation of 2– 8-layer graphene resulted in “craters” consisting of a central hole and a raised circular rim. For 8–14 layers, a removal of the graphene layers, except for the 1–2 bottommost layers, was observed. Due to the circular symmetry of this pattern, it was called “isotropic etching” of the layers. On the thicker layers, estimated at 28 graphene layers (which may be considered bulk graphite), an “anisotropic etching” was observed. In this case, the oxidation process resulted in “fractal-like” patterns with complex and nondeterministic shapes. Atomic force microscopy, Kelvin probe force microscopy (Section 3.2) and Raman spectroscopy (Section 3.3) were employed to characterize and classify all categories of the fabricated graphene structures.

Furthermore, the cases of “by-product” oxidation spreading, observed during traditional area oxidation, are also discussed (Section 3.7). The probability of this phenomenon increased with the number of graphene layers and humidity.

Electric field simulations in COMSOL Multiphysics were performed for 4, 8, and 16 graphene layers containing cleavages of various depths to investigate how the number of layers influences the electric field distribution. The results show that the radial component of the electric field within the graphene layers reaches its maximum when the cleavage penetrates all layers except for the bottommost one. Based on this finding, a theoretical “cleavage model” was developed, qualitatively explaining the formation of the fabricated structures.

## 2 Methods

### 2.1 Experiment

Graphene was prepared by mechanical exfoliation. Graphene flakes were transferred from adhesive tape onto PDMS stamps (surface area ≈ 1 cm^2^). Flakes exhibiting a wide range of layer numbers were selected for further experiments and characterized using AFM topography and Raman spectroscopy. Suitable flakes were subsequently transferred onto silicon (Si) substrates with a 285 nm thick layer of thermally grown insulating silicon dioxide (SiO_2_), equipped with gold electrodes used to ground the graphene during experiments. The graphene layers were positioned such that part of each flake was on the electrode, while the experimental area was beside it. Silver paste was used to connect the electrodes and attach a copper wire connected to the microscope grounding input.

Oxidation spreading experiments were performed using an AFM tip brought into contact with a single point of the graphene under a loading force of approximately 7–15 nN. An oxidation voltage (typically −9 V) was applied to the tip, while the sample was grounded. The relative humidity (RH) of the ambient air was controlled by a humidity chamber within the range of 40–75%, with most experiments carried out at RH values between 60 and 70%. A boron-doped diamond-coated AFM tip (DCP10 NT-MDT) was used for LAO patterning. Experiments were primarily conducted on thicker graphene samples (up to ∼14 nm corresponding to graphite) to understand how the number of layers affects the process and whether this oxidation method can be used for controlled removal of upper layers and monolayer preparation. The exact number of layers at individual experimental locations (6, 8, 9, 12, 14, and 28 layers) was estimated using AFM topography and Raman spectroscopy. First, Raman spectroscopy was used to distinguish between monolayer and multilayer based on the characteristic features of the Raman spectrum. Subsequently, the number of layers was primarily estimated from AFM topography by counting the number of layer steps. In this context, Raman spectroscopy was important to verify the thinnest observed layer.

AFM topography, Kelvin probe force microscopy (using the NTEGRA II microscope, NT-MDT Spectrum Instruments), and Raman spectroscopy (alpha300 R, Oxford Instruments WITec) were employed to characterize the fabricated graphene patterns. The AFM topography was measured in the semicontact (tapping) mode. For the KPFM measurements, the lift height of 10 nm was utilized.

### 2.2 Calculations

Complementary computer simulations were performed to provide insight into the experimental results. The electric potential and electric field between the oxidizing AFM tip and graphene layers were computed using the Maxwell equations for electrostatic problems in COMSOL Multiphysics. The geometry of the simulation, assuming cylindrical symmetry, is shown in Figure 2. The AFM tip was modeled as a sphere with radius *R*, located at a distance *h* from the graphene/graphite surface. Graphene layers were represented by graphite layers 0.3 nm thick (relative permittivity 12), separated by air gaps (*ε*_*r*_ = 1) of the same thickness. Simulations were performed for the structures consisting of 4, 8, and 16 graphene layers, where the central part was removed to simulate material being etched away or the occurrence of a cleavage (Figure 2b). Additional simulations considered 0, 1, 2, 4, 6, 8, 12, and 16 partially removed layers, depending on the initial number of graphene layers.

**Figure 2:**
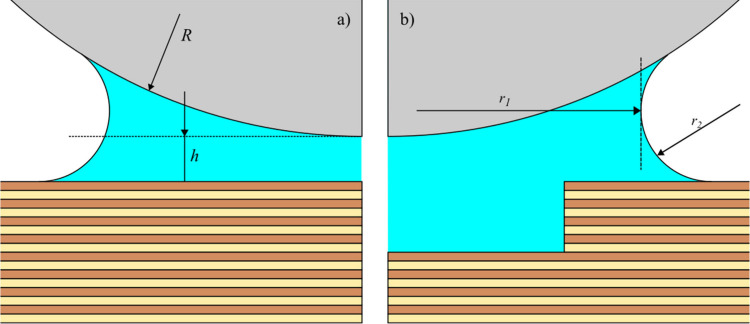
Simulation geometry showing the AFM tip (gray), modeled as a sphere with radius R, positioned at a distance h above (a) compact graphene layers, including layers having properties of bulk graphite (brown) and air gaps (yellow), or (b) partially removed graphene layers, representing material removal or cleavage formation. An approximate water meniscus (blue) characterized by Kelvin radii r_1_ and r_2_ formed between the tip and the graphene.

Ambient humidity (relative humidity, *RH*) leads to the formation of a water meniscus between the tip and the sample. The meniscus shape was calculated using an approximate model using the Kelvin equation and Pythagorean theorem, as described in ref. 23 (Supporting Information), assuming perfect wettability of graphene, such that the water meniscus is tangent to both the tip and the graphene. The meniscus width grows with relative humidity and tip radius, while increasing the tip–sample distance *h* results in narrowing of the meniscus. The relative permittivity of the meniscus is equal to that of water (*ε*_*r*_ = 81). An oxidation voltage (*V* = – 10 V) was applied to the AFM tip, while the bottommost graphene layer was grounded.

## 3 Results

### 3.1 AFM Topography Classification

Approximately 50 LAO experiments were performed using various graphene layer numbers, relative humidities, and exposure times. AFM topography revealed three basic types of fabricated patterns: “graphene craters” (GC), “isotropic etching” (IE), and “anisotropic etching” (AE). The basic character of the pattern was primarily governed by the number of graphene layers, while the pattern radius increased with growing relative humidity.

The topography of GC is shown in Figure 3a, with the corresponding cross-sectional profile presented in Figure 3d. The “graphene crater” consists of a central hole with a radius of approximately 50–100 nm, surrounded by a raised circular rim with a height of about 1–2 nm, and a radius of 150–300 nm. The structure was fabricated on a 6–8-layer graphene. On 6-layer graphene, it formed over the entire investigated humidity range, whereas on 8–layer graphene, it appeared only at relative humidity below 60%.

**Figure 3:**
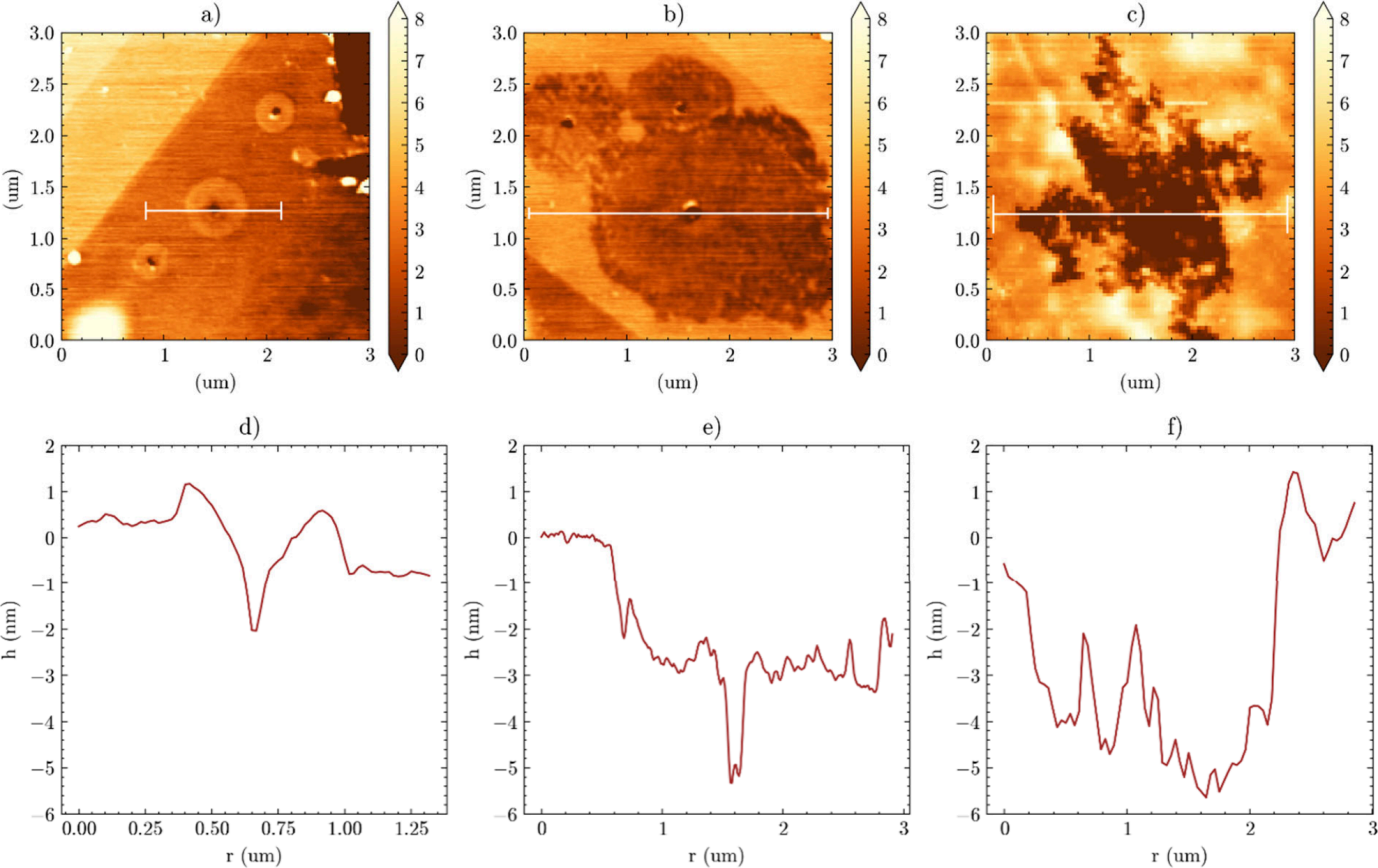
AFM topography of three categories of graphene patterns fabricated by LAO: “graphene craters” (a, d), “isotropic etching” (b, e), and “anisotropic etching” (c, f). The cross-sectional profiles in (d)–(f) follow the white lines in AFM images (a)–(c). The profiles demonstrate that (1) the GC consists of a hole surrounded by lifted graphene, (2) the IE consists of a hole surrounded by an area where the upper graphene layers are removed, and (3) the AE exhibits an irregular shape where the upper graphene layers are removed, with islands of intact graphene scattered across the pattern.

The topography of IE is shown in Figure 3b, with a cross-sectional profile presented in Figure 3e. “Isotropic etching” is a structure where a portion of the graphene layers is removed. The pattern depth typically ranges from 2–4 nm, implying that only the bottommost 1–2 graphene layers remain intact. As suggested by the name “isotropic etching”, the upper layers are removed in a regular, approximately circular shape. In the center of the pattern, a hole penetrating the remaining graphene layers and reaching the substrate is observed. The IE patterns were observed on graphene samples consisting of 8–14 layers, and the pattern radius increased with growing relative humidity.

The topography of AE is shown in Figure 3c, with a cross-sectional profile of the pattern in Figure 3f. Similarly to the IE case, a portion of the upper graphene layers is removed. The pattern depth was measured to be 5–8 nm, implying that the bottommost 1–5 graphene layers remain intact. In contrast to the IE, the pattern shape is irregular; AE patterns typically exhibit “fractal-like” morphology, with islands of intact graphene scattered across the structure. Typically, the patterns have a radius between 500 and 1500 nm. The “anisotropic etching” was observed after the LAO experiment performed on a 28-layer graphene sample (graphite).

The basic characteristics (radius, depth, and surface potential difference, discussed in Section 3.2) of the GC, IE, and AE are summarized in Table 1. The main fabrication conditions for GC, IE, and AE (number of layers and relative humidity) are listed in Table 2. The data show that the GC occurs for a low number of layers and low humidity, while the AE is observed only on 28-layer graphene (as discussed in Section 3.4). The radii of the structures generally increase with increasing relative humidity, as shown in Section 3.5.

**Table 1:** AFM Characteristics of Fabricated Graphene Patterns Divided into Three Categories: “Graphene Craters” (GC), “Isotropic Etching” (IE), and “Anisotropic Etching” (AE)^a^

		GC	IE	AE
Radius (topography) (nm)	Value	200 ± 55	410 ± 330	890 ± 450
	Max/Min	303/150	1275/116	1850/330
Depth (topography) (nm)	Value	–1.1 ± 0.3	3.0 ± 0.9	6.4 ± 1.0
	Max/Min	–0.7/-1.6	4.3/1.3	8.2/4.5
Surface potential difference (mV)	Mean value	20 ± 4	28 ± 16	59 ± 20
	Max/Min	26.3/15.9	66/6	87/24

a The radius, depth, and surface potential difference are generally the highest for AE and lowest for GC. The negative depth value (for GC) indicates the lift of the layers. The surface potential difference is defined as the difference between the KPFM-measured potential of the pristine graphene surrounding the pattern and that of the pattern itself.

**Table 2:** Influence of the number of graphene layers and relative humidity (RH) on characteristics of the fabricated graphene patterns. In each cell, the pattern type (GC, IE, AE) and the pattern radius (nm) are shown. In case the cell is empty, the combination of parameters was not investigated

RH (%)	6 layers	8 layers	9 layers	12 layers	14 layers	28 layers
40–59		GC, 190 ± 20	\	\	\	\
60–65	GC, 240 ± 60	IE, 510	IE, 310 ± 160	IE, 510	IE, 200 ± 140	AE, 970 ± 570
66–71	GC, 170 ± 20	IE, 520 ± 220	IE, 900 ± 380	\	IE, 1200	AE, 760

### 3.2 Surface Potential

The fabricated graphene patterns were characterized using Kelvin probe force microscopy, which measures the surface potential difference between the oxidized pattern and the surrounding pristine graphene. The surface potential map of GC is shown in Figure 4a, with the corresponding profile in Figure 4d. The profile has a parabolic shape, suggesting that the potential is the lowest for the central hole and slightly higher for the lifted circle. The IE (Figure 4b,e) exhibits a constant potential across the etched area. The situation is similar for the AE (Figure 4c,f), where the etched area also exhibits a constant potential, but the complicated shape of the area prevents this constant level from being clearly observed in the profile.

**Figure 4:**
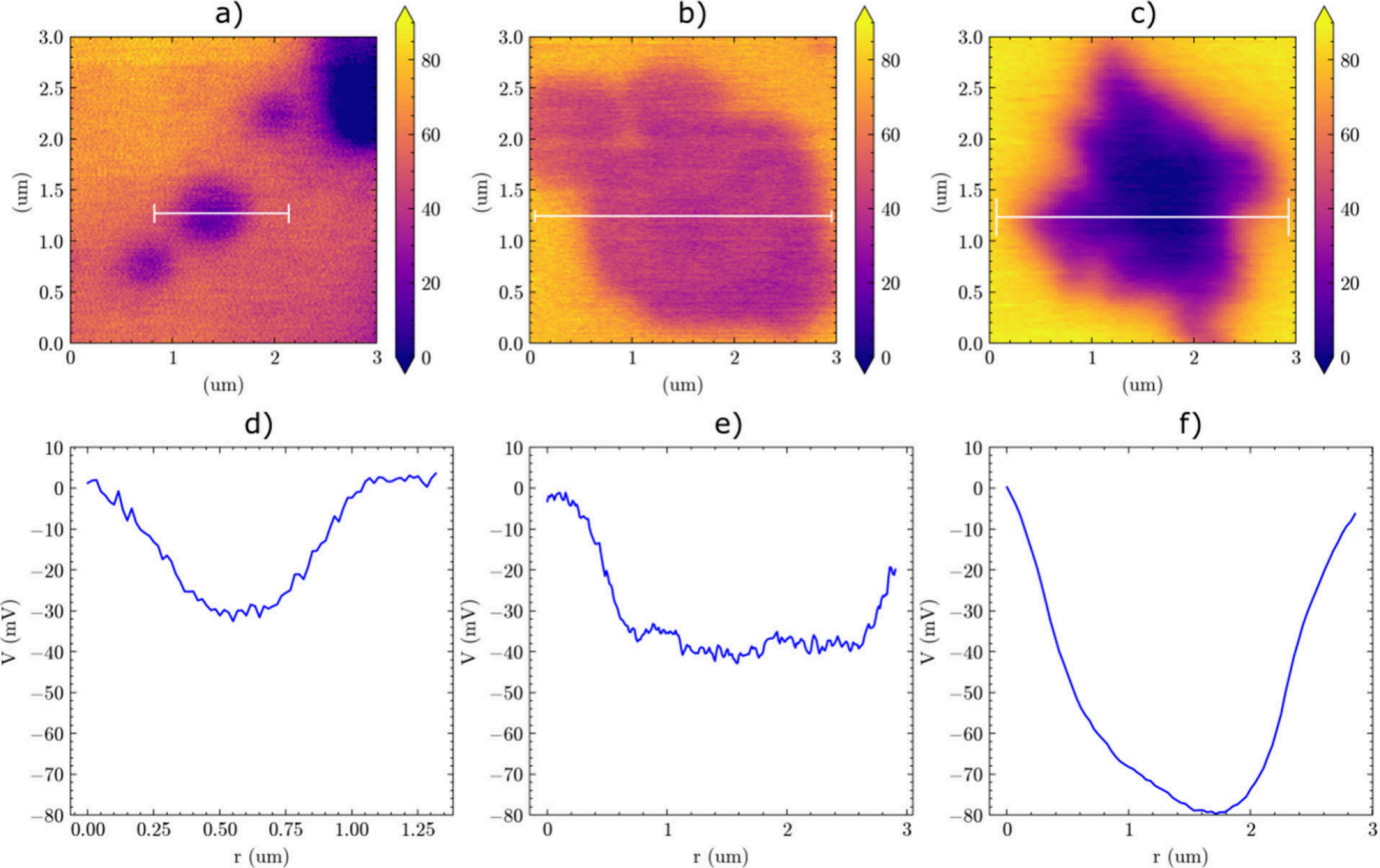
Three types of fabricated graphene structures, shown as KPFM surface potential maps (a–c) corresponding to the topography images presented in Figure 3a–c and corresponding surface potential cross-sectional profiles (d–f): graphene craters (a, d), isotropic etching (b, e), and anisotropic etching (c, f). The cross-sectional profiles (d–f) follow the white lines in the maps, corresponding to the topography cross-sectional profiles in Figure 3(d–f).

An interesting correlation was found between the surface potential difference *V* and the pattern radius *r*. The measured values found for 41 patterns are plotted in Figure 5, with GC shown in green, IE in yellow and AE in red. The data are fitted with an exponential function:

1 $$V=V_{0}\left(1-\exp\left(-\frac{x}{x_{0}}\right)\right)$$

where *V*_0_ = 67.76 and *x*_0_ = 643.69 are the fitted parameters, with a coefficient of determination *R* ^2^ = 0.88.

**Figure 5:**
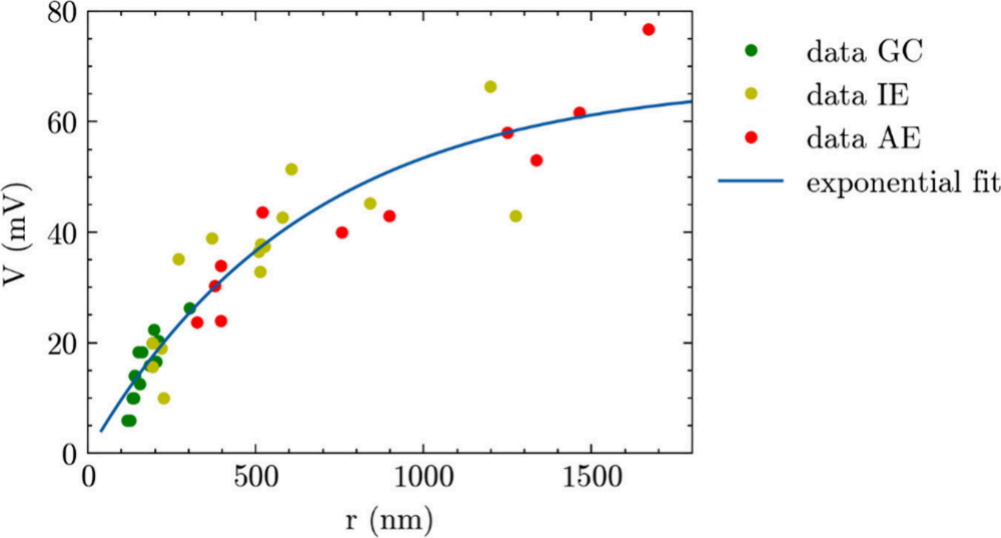
Relationship between the radius r of fabricated graphene patterns and its potential difference (V). Green points: “graphene craters”, yellow points: “isotropic etching”, red points: “anisotropic etching”. The data were fitted with an exponential function V = 67.76 (1 – exp­(−r/643.69), with a coefficient of determination R^2^ = 0.88.

### 3.3 Raman Spectroscopy

The fabricated graphene patterns were characterized by Raman spectroscopy, as shown in Figure 6. For all categories of the patterns (i.e., all combinations of the number of layers and relative humidity), the D peak appeared at approximately 1350 cm^–1^ of Raman shift. For each structure, two spectra were measured – one from the patterned region and one from the surrounding graphene, for comparison. The pristine graphene spectra shown in Figure 6 (blue for 6 layers, cyan for 9 layers, and yellow for 28 layers) exhibit similar features. In particular, the G peak is approximately twice as intense as the 2D peak, which is typical for multilayer graphene. The spectra of “isotropic etching” (9 layers, green) and “anisotropic etching” (28 layers, yellow) both exhibit the D peak slightly more intense than the G peak, while the 2D peak is not apparent. Such spectra are associated with graphene oxide.^3^ The spectrum measured on “graphene crater” (6 layers, red) differs from the previous two cases. Here, the G peak is sharp and significantly more intense than the D peak. The spectrum of the “graphene crater” can therefore be interpreted as a superposition or an intermediate state between pristine graphene and graphene oxide. Two possible explanations can be considered: 1) the laser spot size is large in comparison to the structure size, leading to a measured spectrum that is a superposition of graphene and graphene oxide signals, or 2) due to the lower relative humidity during fabrication, the “graphene crater” was only partially oxidized and therefore exhibits characteristics of both graphene and graphene oxide. The graphene structures shown in Figure 6 were selected as typical examples of the related structures (GC, IE, and AE). Based on this, it is anticipated that the remaining GC, IE, and AE structures underwent oxidation in a similar manner to the demonstrated examples.

**Figure 6:**
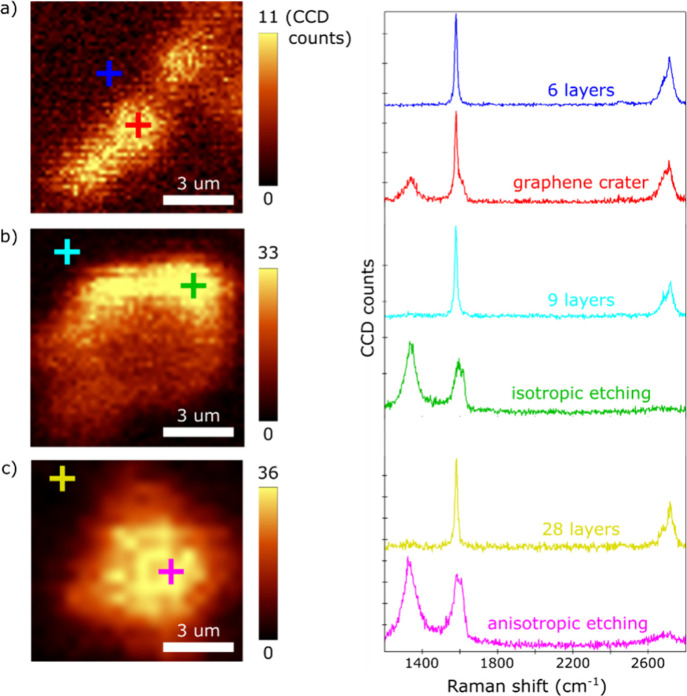
Raman characterization of fabricated graphene patterns. (Left) D-peak (∼1350 cm^–1^) intensity maps. (Right) Raman spectra measured at positions indicated by crosses of the related color. (a) “Graphene craters”, (b) “Isotropic etching”, and (c) “Anisotropic etching”. The graphene structures shown in this figure were chosen as typical representations of GC, IE, and AE.

### 3.4 Number of Layers

The graphene patterns were fabricated on graphene samples with different numbers of layers to investigate the influence of the layer number on the resulting character of the patterns. As mentioned in Section 3.1 and summarized in Table 2, the “graphene craters” were prepared on graphene with 6–8 layers, while the “isotropic etchings” occurred at 8–14 layers, and the “anisotropic etchings” were observed at approximately 28 layers. The influence of the number of layers can be clearly demonstrated on an atomic layer step, i.e., an interface between regions with different layer numbers. Such a step is shown in Figure 7a as a border between regions with six (VI) and eight (VIII) layers. In the 6-layer region, two “graphene craters” with a smaller radius of ∼150 nm and lifted surface (∼0.9 nm) were observed. They were prepared at RH 69%, voltage −9 V, and a loading force of approximately 9 nN. On the 8-layer region, nearly identical conditions were used (RH 68%, voltage −9 V, loading force 8.5–11 nN), resulting in three “isotropic etching” structures with a radius of 250–600 nm, and depth of 1.3–1.9 nm.

**Figure 7:**
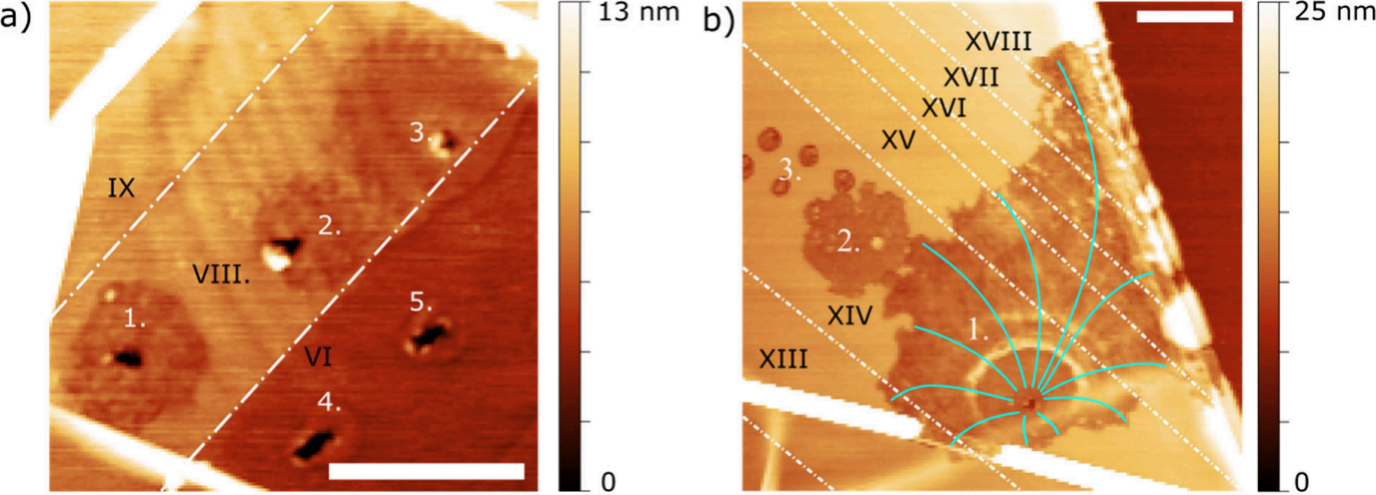
Influence of the number of graphene layers, relative humidity, and exposure time on the character of the patterns fabricated by the LAO of graphene. (a) On the six (VI) graphene layers, “graphene craters” were prepared. On the eighth (VIII) layers, “isotropic etching” occurred. In both cases, the DCP10 tip was used with RH 68–69*%*, voltage of −9 V, and a loading force of 8.5–11 nN.( b) Structure 1 was fabricated by approaching the tip to a point located on the 14 (XIV) graphene layers. On the XIV layers, the oxidation reaction spreads isotropically, as implied by the cyan lines. However, after reaching the region of 15 to 18 (XV–XVIII) layers, the oxidation spreading accelerates with increasing layer number until the flake edge is reached. The influence of humidity and exposure time is also demonstrated: Structure 1 was prepared with RH = 69% and an exposure time of 5 s. Structure 2 was prepared with RH = 64–65% and an exposure time of 5 s. The six structures marked as 3 were prepared with RH = 61–62%, and the exposure time ranged from 5 to 80 s. The white scale bar corresponds to 1 μm.

Structure 1 in Figure 7b was prepared with RH 69%, voltage −9 V, loading force 8.5 nN, and an exposure time of 5 s. The tip was positioned on the 14-layered graphene, from where the cyan lines start, and the oxidation spread isotropically. At the bottom of the image, the reaction was blocked by a wrinkle in the graphene (see the short cyan lines ending at the wrinkle). At the top, the oxidation reaction reached a layer step (to XV layers). At this point, the oxidation reaction accelerated and increased further with every additional layer step up to XVIII. It eventually stopped at the very edge of the graphene flake. As indicated by the cyan lines, the initial isotropic spreading was disturbed by additional graphene layers.

### 3.5 Relative Humidity

While the number of layers significantly influences the character of the structure, the ambient relative humidity relates to its surface area (or radius). Experiments conducted at low humidity levels (below 40%) did not produce any visible structures. When the RH was between 40 and 60%, “graphene craters” were formed with radii ranging from 100 to 200 nm, as shown in Table 2. As indicated there, the radius of GC did not show a clear dependence on humidity. This trend is also illustrated in Figure 8a, where four patterns were prepared with RH ranging from 42 to 63%. Below 60%, only “graphene craters” were fabricated, with radii of 155 nm (42%), 209 nm (52%), and 196 nm (58%). For humidity above 60%, “isotropic etching” occurred with a radius of 510 nm.

**Figure 8:**
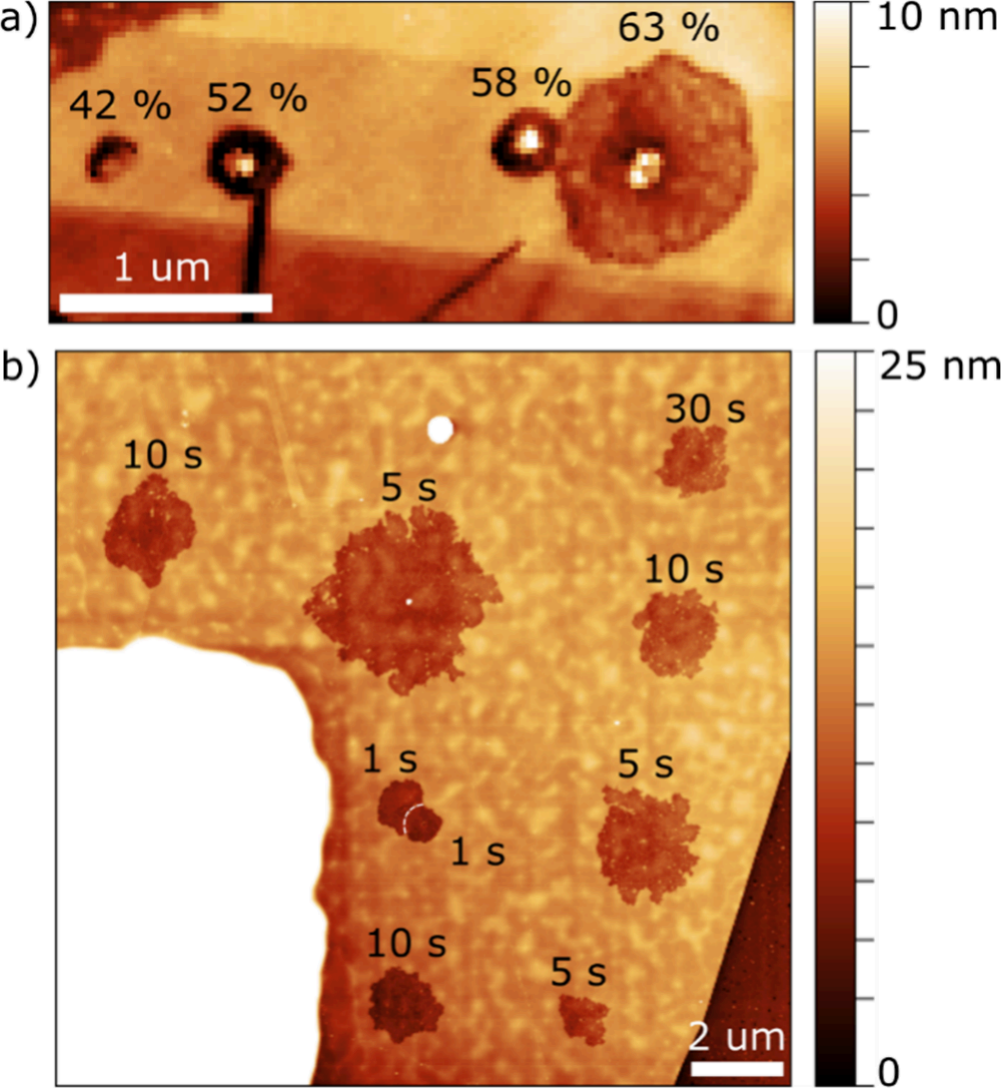
Influence of relative humidity and exposure time on the character of patterns fabricated by LAO on graphene. (a) Four structures prepared with relative humidity values ranging from 40–65%. Below 60% RH, “graphene craters” were observed, whereas above this threshold, “isotropic etching” occurred. (b) “Anisotropic etching” experiments performed with different exposure times, showing no clear dependence of the size of the fabricated structure on the exposure time.

The evolution of “isotropic etching” for relative humidity values ranging from 61 to 69% is demonstrated in Figure 7b. The group of six structures (marked as 3) was prepared using RH = 61–62%, resulting in “isotropic etching” with radii in the range of 125–140 nm. The slightly higher RH of 64–65% resulted in Structure 2, with a radius of 600 nm. For RH = 69%, Structure 1, spreading over 5 layer steps, was prepared, with a radius estimated to be ∼ 1200 nm (measured only on the XIV). All experiments were performed using the same DCP10 tip, with a load force of 7.5–11 nN and a voltage of −9 V.

### 3.6 Exposure Time

No consistent dependence on the exposure time was observed for either thin or thick graphene layers. The six “isotropic etching” experiments shown in Figure 7b (14 layers, RH 61–62%) were conducted with exposure times ranging from 5–80 s, resulting in structures with no significant differences in their radii (the six structures marked as 3). Another experiment was performed on the 28-layer (RH 61–62%, exposure time 1–30 s), resulting in “anisotropic etching” with no apparent dependence on exposure time (see Figure 8b). The experimental results appear somehow counterintuitive, as the largest structures were fabricated with exposure times of 5, 5, and 10 s, whereas some smaller structures were obtained with exposure times of 30, 5, or 1 s.

### 3.7 Oxidation Spreading as a Byproduct

The effect of “oxidation spreading” was also observed as a byproduct of conventional local anodic oxidation (based on scanning over a square or rectangular area). In Figure 9, three examples of this effect are shown. Structure (a) was prepared on 14 nm-thick graphene (estimated to have ∼28 layers) using RH = 65% and a voltage of −9 V, resulting in an area of locally removed graphene. From this hole, “anisotropic etching” spread outward. Structure (b) was prepared on 14 nm-thick graphene, with RH = 75% and a voltage of −9 V, also resulting in “anisotropic etching” spreading from the region of locally removed graphene. Structure c) was prepared on 6 nm-thick graphene (∼7 layers), with RH = 65% and a voltage of −9 V, resulting in “isotropic etching” spreading from an area of partially removed graphene. The revealed SiO_2_ substrate in the center of the structures allows precise comparison of the thicknesses of the pristine graphene and the etched region. The corresponding topography profiles are shown in Figure 9d–f. For structure (a), the thickness decreased from 14 to 6 nm. Structure (b) showed a reduction in thickness from 14 to 4 nm. Structure (c) was thinned from 6 to approximately 1.5–2 nm, indicating that only the bottommost layer was likely preserved.

**Figure 9:**
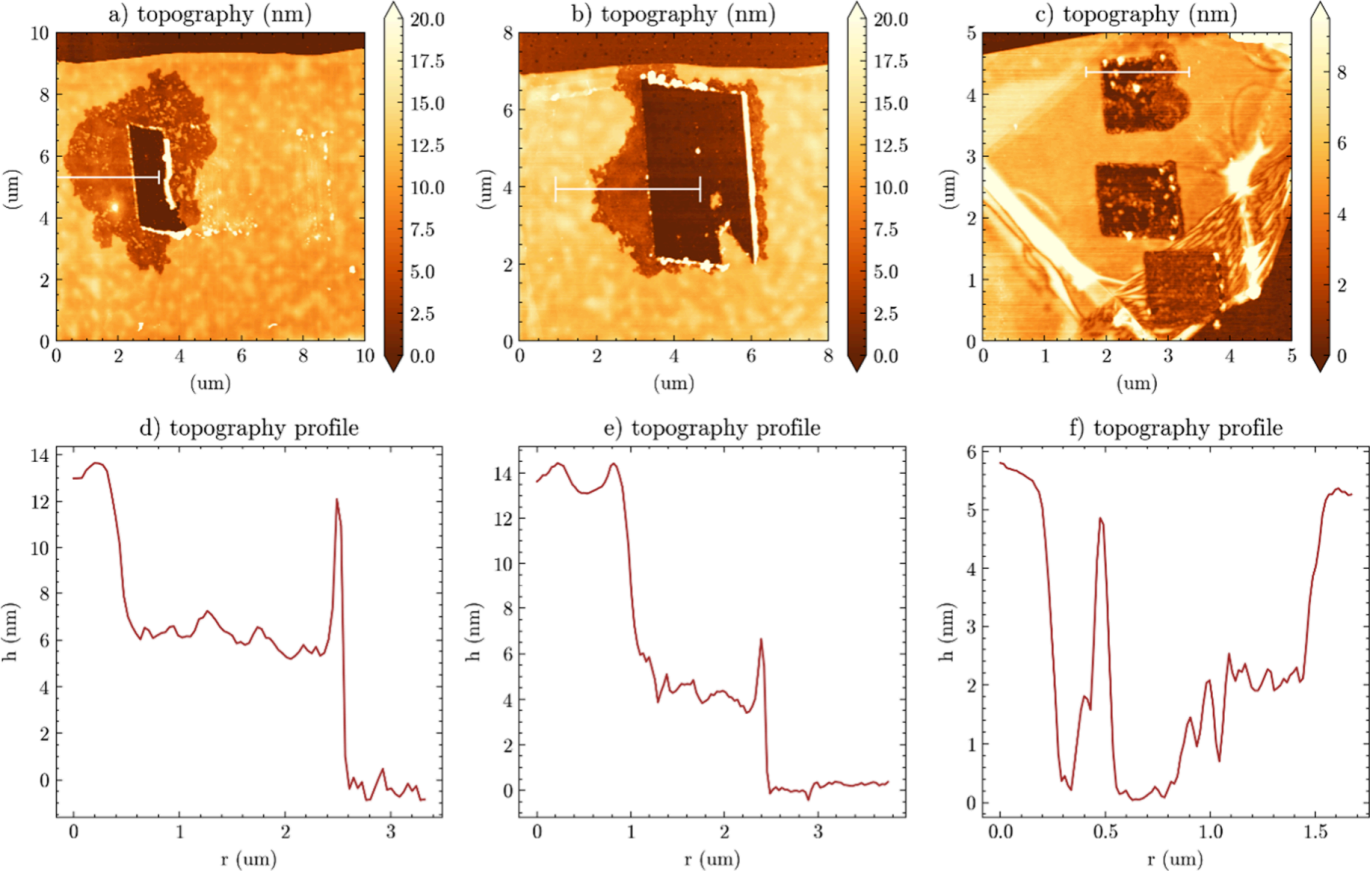
Oxidation spreading as a byproduct: AFM topography images (a–c) and corresponding profiles (d–f) taken along the white lines in panels (a–c). “Anisotropic etching” is observed in (a) and (b), while “isotropic etching” occurs in (c). The layers were thinned from 14 to 6 nm in (a), from 14 to 4 nm in (b), and from 6 to 1.5 nm in (c).

### 3.8 Calculation Results

The electric potential and electric field were computed for the following simulation parameters: *R* = 100 nm (corresponding to the actual tip radius of the DCP10 tip), *h* = 1 nm, *RH* = 67.5%, and *V* = −10 V. The Kelvin radii of the meniscus were taken as *r*_1_ = 16.3 nm and *r*_2_ = 1.27 nm (consistent with ref 23). The graphene layers were removed in a cylindrical space with a radius of 15 nm, as indicated by the dark blue line in Figure 10.

**Figure 10:**
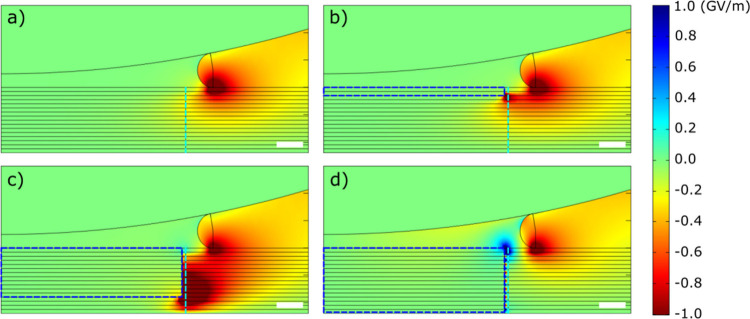
Radial electric field component, E_r_, between an oxidizing AFM tip and multilayer graphene (8 layers) with a water meniscus, calculated using COMSOL Multiphysics. (a) Original state, (b) 1 layer partially removed (dark blue rectangle), (c) 6 layers partially removed, and (d) all 8 layers partially removed. Intensity profiles (see Figure 11) are taken along the light-blue line. The scale bar corresponds to 2 μm.

The radial electric field component is plotted in Figure 10 for an 8-layer graphene system with 0, 1, 6, and 8 layers removed. In all cases, the radial component increases significantly at the bottom of the meniscus. If some of the layers (but not all) are removed under the tip, a second local maximum of the radial field intensity appears in the preserved graphene near the bottom of the cleavage. Profiles taken along the hole edge (at 3 Å, see Figure 11) clearly show the maximum intensity following the bottom of the cleavage. As the number of removed graphene layers increases, the maximum intensity also increases (compare the green, orange, red and purple lines in Figure 11). However, the maximum disappears when all graphene layers are locally removed, with no pronounced radial intensity present at the hole edge. Instead, the profile exhibits local minima and maxima at the boundary between the graphene and intercalated air layers (see the black line in Figure 11).

**Figure 11:**
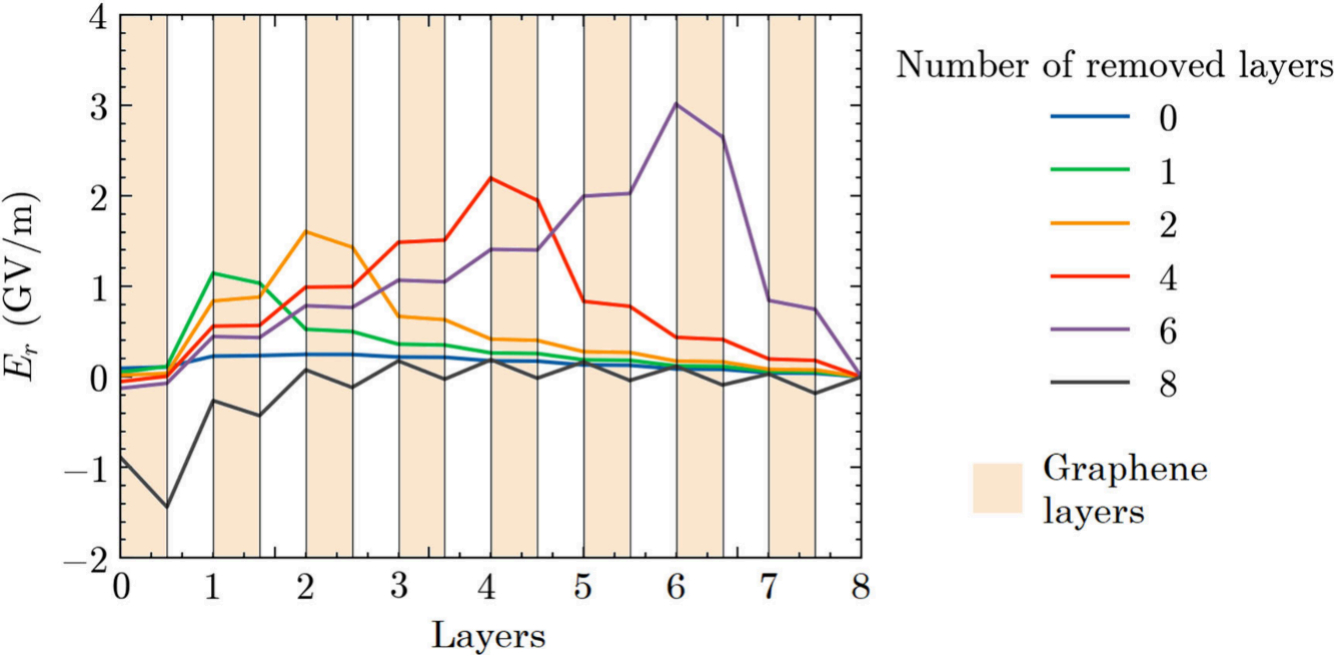
Profiles of the radial component of the electric field (E_r_) in graphene layers close to (3 Å from) the area of removed layers. For all profiles except when all 8 layers are removed, the maximum intensity occurs at the bottom of the cleavage. The maximum radial intensity increases with the number of removed layers.

In Figure 12, the maximum radial electric field intensity values for various numbers of graphene layers and the corresponding numbers of removed graphene layers are listed. For comparison, cases with 4, 8, and 16 graphene layers were calculated. The maximum always occurs for one persisting layer (while the others are removed). The maximum slightly decreases with an increase in the number of layers.

**Figure 12:**
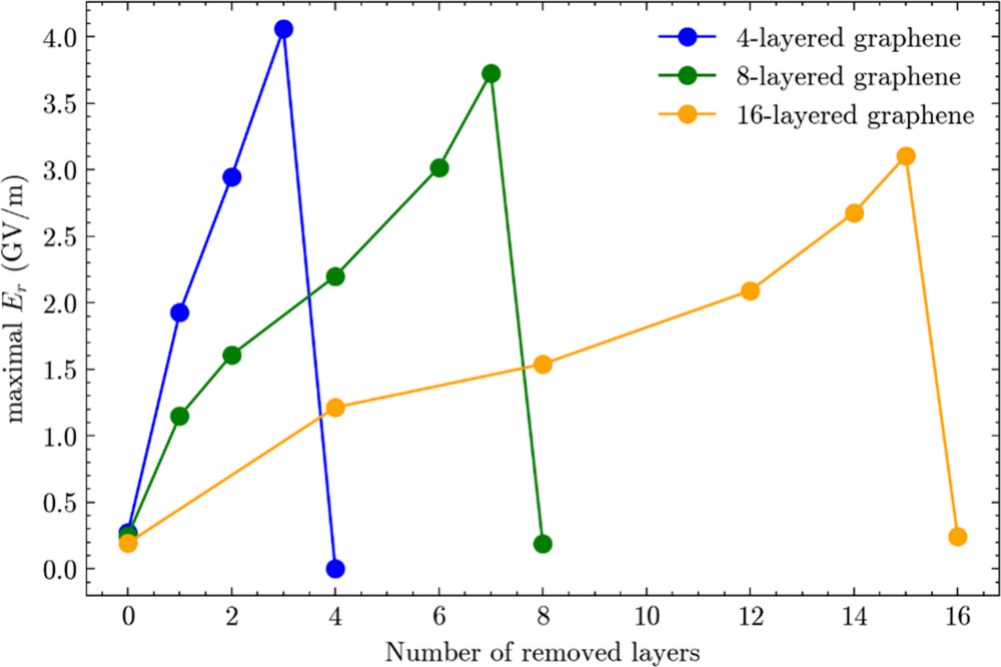
Dependence of calculated maximum radial electric field intensity along the cleavage edge on the original number of graphene layers and the number of removed graphene layers. The maximum intensity slightly decreases with the number of graphene layers and significantly increases with the number of removed graphene layers.

## 4 Discussion

The results of the electric field calculations suggest that the radial component of the electric field, responsible for the movement of the oxygen ions between the graphene layers, reaches its maximum value at the bottom of the cleavage when the cleavage penetrates through all layers except the bottommost one. The experimental results are consistent with the calculations, as the AFM topography shows that both the “isotropic” and “anisotropic” etching remove a large portion of the graphene layers, leaving only 1–2 (“isotropic”) or 8–12 (“anisotropic”) of the bottommost layers intact. It is important to note that the “anisotropic etching” was observed only in the case of very thick graphene layers (14 nm, estimated as 28 layers), and the oxidation process could not remove all of them.

Based on the experimental and calculation results, a qualitative “cleavage model” was proposed. It assumes that some density of defects is inherently present in the graphene in a nonhomogeneous manner. Certain parts of the graphene flake are more defective than others. Some of the defects take the form of cleavages that penetrate multiple graphene layers, sometimes even all of them. When the AFM tip is approached, a negative bias voltage is applied between the tip and the graphene, and a water meniscus forms. The exposed surface and cleavages are available for reaction with negative oxygen ions produced by electrolysis in the meniscus. The electric field drives the ions into the cleavages and subsequently into the space between the graphene layers. This results in the disruption and removal of the layers. This “etching process” may initiate at each cleavage near the tip. However, as previously noted, the radial intensity is strongest for the deepest cleavages. Therefore, the reaction spreading from cleavages that penetrate all layers except the bottommost one is expected to be the most intense. Ultimately, all affected areas would be “etched” by a reaction emanating from a single cleavage, leading to an “isotropic etching”.

In the case of “isotropic etching”, a hole going through the graphene layers was observed in the AFM topography. This hole, probably formed mechanically by the AFM tip, is the most probable source of the reaction. In the case of the “graphene crater”, the relative humidity of the environment was too low to enable meniscus formation, so the reaction could not begin. Because of that, the Raman spectra of “craters” are different from those of “etched structures” and more similar to pristine graphene.

In the case of the “anisotropic etching”, the tip could not penetrate all the graphene layers; the 14 nm layer was too thick to enable this. Consequently, the reaction spreading from the “tip-initiated” cleavage was not as intense as in the case of IE. Thus, the tip-initiated-cleavage reaction did not fully dominate the oxidation process, and the reaction spreading from other local defect-based cleavages resulted in a complex, nondeterministic shape of the fabricated structure.

In the case of large “anisotropically etched” structures, the oxidizing reaction diminishes its intensity with growing distance from the tip. The number of remnants (small areas of nonetched graphene layers) is growing with the structure radius. It is possible that for the higher radii, the “etching process” can remove only defective parts of the graphene layers, so the undefective parts are preserved in the form of these remnants.

The reaction would spread rapidly and may stop when the radial intensity decreased below the threshold required to sustain etching. After reaching this threshold, prolonged exposure of graphene to the oxidizing voltage does not further affect the resulting structure. This would explain the apparently chaotic experimental results related to exposure time (Section 3.6). The size of the structure would be determined by the interaction between the atoms of the tip and the graphene surface (i.e., cleavage created by the tip) and by the local density of the defects.

## 5 Conclusion

Local anodic oxidation of graphene layers is typically performed by scanning the area determined for modification. Here, an alternative approach was used, based on an approach of the AFM to a single point of the graphene, with negative bias voltage applied between the tip and the graphene, allowing the oxidation reaction to spread from the tip into its surroundings. This way, three types of structures were fabricated on the graphene multilayer. Low humidity (under the threshold allowing the formation of a water meniscus) and a low number of layers resulted in relatively small “graphene craters”, while higher values of these parameters resulted in “isotropic etching” or “anisotropic etching”—the removal of almost all the graphene layers. The experimental results can be explained by the electrostatic calculations performed in COMSOL Multiphysics, which show that the maximum radial electric field occurs at cleavages penetrating all graphene layers except the bottommost one. Using both experimental and calculated results, a qualitative “cleavage” model was proposed to explain the reaction and formation of the fabricated structures.

## Data Availability

The data supporting the findings of this study are openly available in Zenodo at 10.5281/zenodo.19707666.
